# Tomato as Potential Source of Natural Additives for Meat Industry. A Review

**DOI:** 10.3390/antiox9010073

**Published:** 2020-01-15

**Authors:** Rubén Domínguez, Patricia Gullón, Mirian Pateiro, Paulo E. S. Munekata, Wangang Zhang, José Manuel Lorenzo

**Affiliations:** 1 Centro Tecnológico de la Carne de Galicia, Rúa Galicia No 4, Parque Tecnológico de Galicia, San Cibrao das Viñas, 32900 Ourense, Spain; rubendominguez@ceteca.net (R.D.); patriciagullon@ceteca.net (P.G.); mirianpateiro@ceteca.net (M.P.); paulosichetti@ceteca.net (P.E.S.M.); 2 College of Food Science and Technology, Nanjing Agricultural University, Nanjing 210095, China; wangang.zhang@yahoo.com

**Keywords:** tomato by-products, reformulated meat products, carotenoids, lycopene, extraction techniques, natural additives, antioxidant, colourant

## Abstract

Tomato industry produces huge amounts of by-products that represent an environmental and economic problem. However, these by-products contain multiple bioactive compounds, which would make them a renewable source for obtaining natural antioxidants and colourants (carotenoids). This is in line with the preferences of the current consumer who demands more natural and healthy products. However, the lipophilic character of carotenoids means that their extraction must be carried out using toxic organic solvents. To overcome environmental and health problems of organic solvents, the application of supercritical fluid extraction (SFE) for the extraction of lipophilic compounds such as lycopene was used successfully, achieving yields similar to those obtained with conventional techniques. Nonetheless, the extraction conditions must be carefully selected, to obtain high yields and at the same time maintain a high antioxidant capacity. On the other hand, the use of tomato and tomato extracts as natural additives in meat products are reduced in comparison with other natural antioxidant/colourant extracts. However, different researches conclude that the use of tomato improved nutritional quality, reduced lipid oxidation and increased stability during the shelf-life period of meat products, while retaining or increasing sensory properties and overall acceptability, which converts tomato by-products into a promising source of natural additives.

## 1. Introduction

Lipid oxidation is the main non-microbial cause of quality deterioration of meat and meat products. Oxidative reactions reduce nutritional value of meats, produce several toxic compounds that can promote multiple diseases and reduce their sensory quality [1]. To this regard, colour is the most important parameter that influences consumer acceptance [2].

With this in mind, the main strategy used by the meat industry to inhibit lipid oxidation is the addition of antioxidants to meat and meat products [1]. Furthermore, in order to maintain the acceptable colour, some additives as nitrite or colourants could also be added to meat. However, several studies indicated a relationship between synthetic additives intake and some health issues [3] leading to increased consumer demands for more natural products. This fact limits the industry in their use of synthetic additives in foods, leaving manufacturers with few options [4]. Due to everything mentioned above, there is a growing interest in the use of new techniques in food processing, re-formulated products and replacing synthetic additives by natural bioactive compounds [5,6,7,8,9], as well as the use of active packaging [7]. Additionally, special properties (anti-inflammatory and/or antioxidant) of some natural additives lead to potential beneficial health effects [10], which determine the preference of consumers for naturally derived antioxidants and colourants because they are associated with healthy and good quality products [11]. Among all-natural pigments, carotenoids present in several plants and fruits are promising additives, which possess important antioxidant activity and intense colour. Therefore, they can be used as potent antioxidants and colorants in the food industry [3].

On the other hand, in the context of a circular economy, efforts are being dedicated to the use of natural additives from by-products generated by the agro-food industry and from underexploited plant materials [3]. Natural additives also have the advantage of being readily accepted by consumers [5]. One of the agro-food industries, which produces a large number of by-products is the tomato industry. These by-products consist mainly in a mixture of tomato peels, pulp residues and seeds that account 7–7.5% of raw materials [12]. The tomato by-products remain unutilized, and they not only add to the disposal problem, but also aggravate environmental pollution. One way of avoiding these problems would be to reuse the tomato by-products, which represent a renewable source that contains large quantity of potentially bioactive compounds [13,14,15]. Thus, both, the quantity of by-products generated during tomato processing and potential bioactive compounds justifies the great interest in extracting carotenoids from tomato by-products [16]. 

Tomato by-products are rich in multiple compounds with antioxidant and colourant properties such as carotenes (lycopene, β-carotene, phytoene, phytofluene and lutein), phenolic compounds (phenolic acids and flavonoids), vitamins (ascorbic acid and vitamin A) and glycoalkaloids (tomatine) [13,17,18]. Among them, lycopene is the most important bioactive compound present in the ripened tomato (80–90% of the total pigments) [9]. Additionally, several epidemiological reports evidenced the health benefits derived from carotenoids [18] and specially lycopene that is the most promising carotenoid for implications associated with human nutrition and health [19]. To this regard, it was reported that the bioactive compounds derived from tomato and tomato by-products have anti-inflammatory, antiallergenic, antimicrobial, vasodilatory, antithrombotic, cardioprotective and obviously, antioxidant and colorant properties [14]. Moreover, multiple studies concluded that a diet rich in tomato and tomato products possesses potential health benefits [11], such as a decreased the risk of occurrence of cardiovascular diseases and various types of cancer [20].

On the other hand, the extraction procedures to obtain carotenoids from multiple plants and fruits involve the use of toxic organic solvents, which may be present at trace level in the final extract. Therefore, in order to obtain a “clean” extract from tomato by-products and limit the environmental impact of its obtaining, environment-friendly extraction methodologies must be used [14]. This fact allows to convert tomato by-products into new food ingredients or natural additives [12].

**Figure 1:**
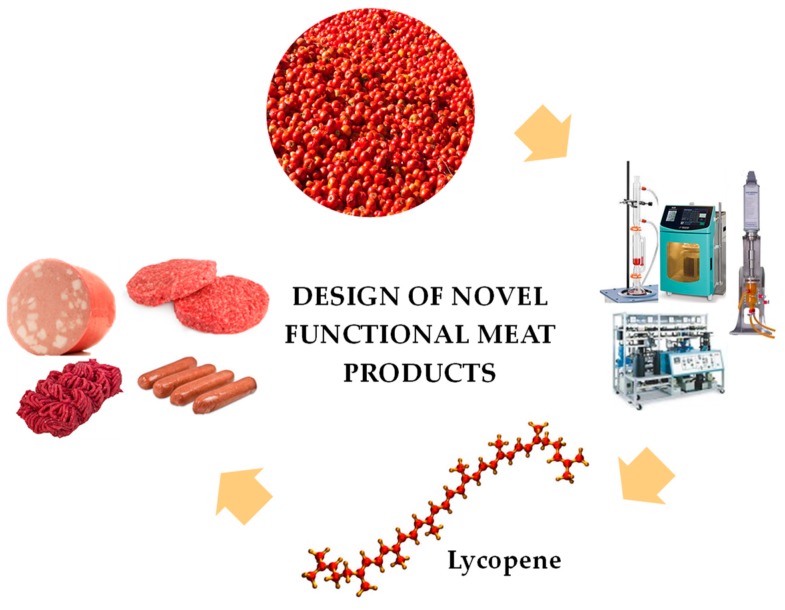
Overview of lycopene extraction technologies from tomato by-products and their application in meat products.

Considering the excellent antioxidant properties and the intense red colour of tomato and specially, lycopene, they could be used in meat industry in order to prevent oxidative and discolouration degradation and the production of a “functional” food, enriched in lycopene which brings multiple health benefits. These aspects are highlighted in Figure 1.

Several reviews on natural antioxidant sources have been published in the last decade. However, this paper is specially focused on the characterization of the bioactive compounds of tomato by-products, the main techniques to extract carotenoids and the use of tomato by-products or their extracts as natural additives in meat industry.

## 2. Bioactive Compounds Present in Tomato

Tomato is one of the most globally-consumed vegetables, being a key component of the Mediterranean diet [13]. Tomatoes, tomato-based products, as well as the by-products generated in their processing are an excellent source of phytochemicals, including carotenoids (mainly lycopene and β-carotene), polyphenols (phenolic acids and flavonoids), vitamins (ascorbic acid, tocopherols and vitamin A), glycoalkaloids (tomatine) and minerals (K, Mn, Ca, Cu and Zn) [21]. These bioactive constituents are recognized by their health benefits, namely anticarcinogenic, cardioprotective, antimicrobial, anti-inflammatory, antioxidants properties, among others [13]. It is important to highlight that the content of bioactive compounds depends on tomato variety, agricultural practices, environment conditions, ripeness, as well as the processes of industrial transformation of tomato into juices, ketchup, pastes, purees, sauces and soups [22].

### 2.1. Carotenoids

Carotenoids are a group of over 750 natural pigments synthesized by plants, bacteria, fungi and some algae. These pigments provide the yellow, orange and red colours of many fruits and vegetables. Carotenoids may be divided into two groups according to the differences in the structure of the polyisoprenoid chain: carotenes, which are hydrocarbon carotenoids that are either cyclized (α-carotene and β-carotene) or linear (lycopene) and xanthophylls that contain one or more oxygen molecules (lutein, zeaxanthin, astaxanthin and canthaxanthin) [23]. Carotenoids cannot be synthesized by humans, so they must be incorporated through diet, being tomatoes and tomato-based foods the main dietary sources of carotenoids available [24]. Additionally, the tomato processing by-products also present significant amounts of carotenoids so they could be used for the development of functional foods.

#### 2.1.1. Lycopene

Lycopene is the major carotenoid present in ripe fruits of tomato, accounting for approximately 80–90% of these pigments [21]. Regarding its molecular structure, lycopene consists of a 40-carbon-atom chain with 11 conjugated and two unconjugated double bonds [24]. This distinctive structure explains its red coloration, as well as its lipophilic character. Due to its linear structure and lack of a β-ionone ring, lycopene has no pro-vitamin A activity [24]. It is present in foods mainly as all-trans-isomer, which is the most stable isomer thermodynamically [25]. However, thermal processing induces isomerization of the all-trans isomer to the cis-form, which is more bioavailable for humans [24]. 

Regarding the intake of lycopene, at least the 85% comes from the intake of tomato-based products [21]. The amount of lycopene in fresh tomatoes and tomato products presents a high variability depending on variety, maturity, geographical site of cultivation and type of processing [24]. In ripened tomato, lycopene is present in amounts ranging from 1.9 to 6.5 mg/100 g FW [26]. In processed tomato products the concentration of lycopene is much higher; for example, in tomato concentrates is of 54 mg/100 g, in ketchups reaches 16.6 mg/100 g and sauces achieves 20.86 mg/100 g [24].

The intake of lycopene has multiple health benefits that have been well documented. For example, dietary lycopene exhibits important bioactivity in the prevention and therapy of cardiovascular diseases [24]. Several epidemiological evidences have related higher lycopene consumption with reduced prostate cancer risk [27]. Due to its antioxidant properties, and its lipophilic nature, the lycopene has also been investigated for its potential role in the prevention of lesions caused by oxidative stress at the brain level [28]. Moreover, other authors have confirmed the beneficial effects of a lycopene-rich diet on the pathogenesis of osteoporosis [29]. 

#### 2.1.2. β-Carotene

β-carotene is the second most abundant carotenoid found in tomatoes and is responsible for the yellow and orange coloration. Depending on the tomato variety, mean levels of β-carotene are in the range 0.23 and 2.83 mg/100 g of FW [30]. As occurring with lycopene, some studies have reported that tomato processing by-products contain higher significant amounts of β-carotene than in whole tomatoes (14.9 vs 8.6 mg/100 g DW) [31]. β-Carotene contains two retinyl groups, being the main precursor of Vitamin A. In the intestine epithelium, β-carotene is converted to retinol by the enzyme 15,15′-oxygenase [32].

Besides the pro-vitamin A activity, some epidemiological studies also confirm other biological activities of β-carotene, including antioxidant capacity, improvement of the immunological function, prevention of several types of cancer and cardiovascular disease [15]. Despite these positive effects, the intake of β-carotene supplements in high doses, especially in smokers, could increase the incidence of lung cancer [15].

### 2.2. Phenolic Compounds

Phenolic compounds are one of the main phytochemicals present in both fruits and vegetables and the by-products generated in their processing. The phenolic compounds in raw tomatoes, tomato products and by-products from the tomato processing include flavonoids (rutin, naringenin, naringenin chalcone, kaempferol and quercetin) and phenolic acids (hydroxycinnamic, chlorogenic, p-coumaric, ferulic and caffeic acids) [33]. Both the content and the profile of phenolic compounds are significantly influenced by the tomato variety, as well as by the part of the fruit considered [33]. To this regard, a study found that the amount of total phenolic in cherry tomatoes ranges between 64.6 and 440.0 mg/100 g DW [34] while the levels of total polyphenols in different commercial and wild/exotic cultivars of tomato varied between 26.34 to 66.08 mg gallic acid equivalent (GAE)/100 g FW and 62.82 to 141.98 mg GAE/100 g FW, respectively [35]. Among the main phenolic compounds identified, it was reported that the naringenin chalcone was the most abundant (309.7 mg/100 g DW), followed by 3-caffeoylquinic acid (71.1 mg/100 g DW) and quercetin-3-rutinoside (60 mg/100 g DW) [34]. A more recent research also found important differences in the phenolic profile between different tomato cultivars [36]. Rutin was identified as the main flavonoid with concentrations in the range of 1.24–3.63 mg/100 g FW followed by naringenin (0.65–1.19 mg/100 g FW), quercetin (0.048–0.141 mg/100 g FW) and myrcetin (0.017–0.286 mg/100 g FW). Chlorogenic acid was the most abundant phenolic acid and ranged from 0.75 to 1.38 mg/100 g FW. 

Several studies have also highlighted remarkable differences in the content of phenolics between the diverse fractions of tomato fruit [22]. Research was carried out to determine the total phenolic content in different fractions (skin, seeds and pulp), and found that tomato skin and seeds presented higher amounts of polyphenolics than pulp (29.1 and 22 respectively, vs. 12.7 mg GAE/100 g FW) [37]. The same observation has also been made by others, who related that the phenolic content of several tomato types (grape, cherry, bola and saladette type) was on average 2.2 times higher in the skin than in the seeds [22].

In addition, the content of phenolic compounds could also be affected by mechanical and thermal treatments during industrial tomato processing. In this context, there are conflicting data on the stability of these bioactive compounds during the process of tomato-based products. Some authors observed an increase in various flavonoids in the tomato sauce processing as compared to the fresh tomatoes [38]. Specifically, they reported an increase of 7-fold in the flavanone naringenin, 4-fold in the protocatechuic acid and 3-caffeoylquinic acid while the rutin level was increased 2-fold. Similarly, another study also reported that the processing of tomato fruit into sauce resulted in both an increase in naringenin (20-fold higher) and an improvement in antioxidant activity (1.2-fold higher) [39]. However, a significant decrease in total polyphenol content during the manufacture of tomato puree was also observed by a different research [40].

In recent years, the interest in dietary phenolics has increased due to its numerous health-promoting properties. In this context, epidemiological evidence suggests that the consumption of fresh tomatoes and tomato products is associated with the prevention of a large variety of diseases such as cardiovascular disease, alzheimer’s or certain types of cancer [41]. Furthermore, many of these beneficial effects have also been reported for by-products generated during tomato processing. A study confirmed the antiproliferative activity of bioactive phenolic extracts from tomato wastes in three cell lines, namely HeLa (cervix epitheloid carcinoma), MCF7 (breast adenocarcinoma) and MRC-5 (fetal lungs) [33]. Additionally, other research demonstrated that phenolic compounds from the peel and seeds of different tomato varieties possessed antimutagenic activity [22].

### 2.3. Vitamins

Tomato is a magnificent source of vitamin C and has important levels of vitamin A, B and E. Concerning vitamin C (ascorbic acid), tomato represents one of the main sources of this vitamin in the Mediterranean diet. Ascorbic acid is thermally labile and light-sensitive and can be easily degraded during the thermal processing and storage of the food [42]. The vitamin C degradation has been considered in various works, who reported a remarkable loss of this vitamin (approx. 80–90%) after the pasteurization of tomato puree [40,43]. The vitamin C in tomato can be found at concentrations ranging from 8.0 and 16.3 mg/100 g FW, depending on the genotype, fruit development and environmental conditions [36]. It is a water-soluble vitamin that is easily absorbed in the body but it is not stored and is required for multiple biological functions [44]. Its health-promoting effects are related to its ability to act as an electron donor, being a potent antioxidant that protects lipid membranes and proteins from oxidative damage [44]. In fact, vitamin C can prevent low-density lipoprotein (LDL) oxidation acting as antiatherogenic [45]. This vitamin has important beneficial effects for the skin, since it is an essential cofactor for the two enzymes required for collagen synthesis. Recently, the role of vitamin C has also been reported to ameliorate neurodegenerative diseases [46].

In turn, vitamin E is a fat-soluble compound with remarkable antioxidant activity that includes eight different chemical structures with four tocopherols (α-, β-, γ- and δ), and four tocotrienols (α-, β-, γ- and δ). These molecules only differ in their aliphatic tail; the tocopherols possess a phytyl side chain linked to their chromanol nucleus, while the tail of tocotrienols presents three double bonds forming an isoprenoid chain [47]. It has been reported that these forms of vitamin E have different biological activities. Although humans absorb all forms of vitamin E, only the α-tocopherol is maintained in human plasma and is used to define recommended dietary allowances of vitamin E [48]. Vitamin E is an essential nutrient that cannot be synthesized by the human body and therefore must be provided through the diet [23]. In this regard, tomato is an important source of vitamin E. The tocopherol content in tomatoes is in the range of 0.17 to 1.44 mg/100 g FW [49]. 

Numerous studies have recognized the role of vitamin E to human health and disease prevention. Most of the known functions of this vitamin are attributed to its excellent antioxidant capacity that inhibits the formation of reactive oxygen species molecules when fat undergoes oxidation during the propagation of free radical reactions [50]. Vitamin E plays a key role in the maintenance of skeletal muscle homeostasis and promotes plasma membrane repair [51]. Other studies showed that this vitamin could decrease the risk of type-2 diabetes, improve cardiovascular functions [50] and reduce the risk of prostate cancer [52].

### 2.4. Glycoalkaloids

Glycoalkaloids are secondary metabolites present in the *Solanaceae* family. These metabolites play a major role in the protection against phytopathogens and may exhibit important biological functions in animals and humans. In tomatoes, these glycoalkaloids are present in the form of tomatine and esculeoside A [21,53]. Tomatine, in particular, consists of a mixture of α-tomatine and dehydrotomatine. The highest levels of tomatine are found in green tomatoes (500 mg/kg FW), while in ripe red tomatoes the content of this glycoalkaloid decreases (5 mg/kg FW). In turn, the levels of esculeoside A are higher in the ripe fruit, varying from 9 to 53 mg/100 g FW [54]. The content of both glycoalkaloids is influenced by cultivar type and agronomic factors. Several studies have suggested that both glycoalkaloids possess numerous beneficial health effects such as anti-cancer activity, ability to reduce low-density lipoprotein cholesterol and triglyceride levels, the stimulation of the immune system and protection against bacterial and protozoa [53]. For example, it was demonstrated that α-tomatine presented high bioactivity against prostate cancer cells [55] and is a potent growth inhibitor of human colon (HT29) and liver (HepG2) cancer cells [56]. In that study, the authors highlighted that tomatine at a concentration of 1 μg/mL exhibited greater anti-cancer activity against human liver cancer cells than observed with the commercial anticancer drug doxorubicin. Esculeoside A and tomatine have also been exhibited the ability to inhibit breast adenocarcinoma cells proliferation [57]. Additionally, it was found that the intake of tomatine in mice led to a reduction in serum cholesterol, LDL cholesterol levels and ameliorated the severity of atherosclerotic lesions [53]. Similarly, esculeoside A also showed the ability to ameliorate hyperlipidemia and aterosclerosis [58] and block hyaluronidase activity and ameliorate the symptomatology of atopic dermatitis [59]. 

## 3. Carotenoids Extraction Techniques

**Figure 2:**
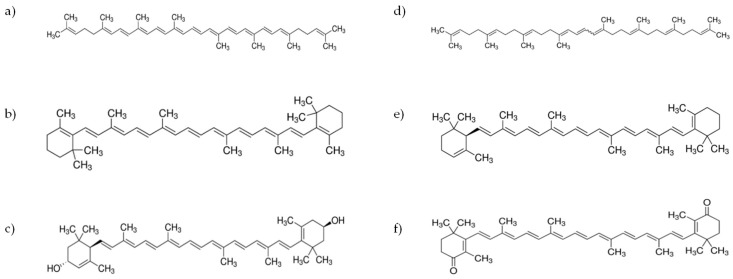
Chemical structure of various carotenoids that are present in tomato and tomato by-products; (**a**) Lycopene, (**b**) β-carotene, (**c**) lutein, (**d**) phytoene, (**e**) α-carotene, (**f**) canthaxanthin.

Several carotenoids were found in tomato and tomato by-products. As commented throughout this manuscript, lycopene is the most important carotenoid and represent about 88% of total carotenoids, followed by β-carotene, phytofluene and phytoene with similar amounts (2–3% each) and lutein (≈1.5%). The other carotenoids represented less than 1% [60] (Figure 2). Despite this, as commented above, the content of carotenoids in tomato depends on several factors including cultivars, soil and climate conditions, degree of ripening and post-harvest storage conditions [13,61]. Considering that carotenoids are the main bioactive compounds in tomatoes, the published studies carried out with tomato focus mainly on the extraction of carotenoids, or more specifically lycopene. Therefore, this section discusses the different carotenoid extraction techniques used in tomato and tomato by-products.

Extraction efficiency is determined by the structure of the individual carotenoids. Xanthophylls are more soluble in hydrophilic solvents, whereas carotenes possess a more hydrophobic nature, which limits their solubility in water and has high solubility in non-polar solvents [16].

Lycopene is insoluble in water, barely soluble in ethanol, while it presents high solubility in lipids and non-polar organic solvents [62]. Thus, both, lycopene and other carotenoids are usually extracted employing organic solvents and also industrially produced by chemical synthesis [13]. Since these processes involve the use of highly toxic chemical solvents, interest has grown in the use supercritical fluid extraction (SFE) as solvent alternative to the industrial production of lycopene [13]. Extracts obtained using this technology has the advantage that does not contain residual solvent [18]. After extraction and solvent removal, a semisolid mixture of resin and essential oil (called oleoresin) is obtained. This oleoresin is rich in carotenoids, however the carotenoids and lycopene amounts depend on several factors as their initial amount in the raw material and the extraction conditions [63].

### 3.1. Conventional Techniques: Organic Solvent Extraction

Several organic solvents, such as ethanol, acetone, petroleum ether, hexane, benzene, chloroform itself or in their combinations were used for extraction of lycopene from tomato or tomato by-products [64]. In general, solvent mixtures containing a polar and a non-polar component as hexane/acetone, hexane/ethanol or hexane/acetone/ethanol were suggested as the best solvent systems for extraction of both, polar and non-polar carotenoids from vegetables [16,65]. Moreover, the use of hexane, acetone, ethanol and methanol and their mixtures is better than the use of other solvents as diethyl ether and tetrahydrofuran, which may contain peroxides that react with carotenoids [66], while the stability of lycopene is higher in hexane/acetone or hexane/ethanol extracts than in extracts obtained with chloroform, methanol or dichloromethane [67].

However, not only the solvent composition or polarity affect the carotenoids extraction. In fact, other parameters, as extraction temperature, particle size, solid/solvent ratio or the use of auxiliary technologies have great importance in the carotenoids’ extraction. Table 1 shows the solvents and extraction conditions used to extract lycopene and carotenoids from tomato by-products. 

A research that tested different individual organic solvents found that ethyl lactate was the most efficient solvent in the lycopene recovery (243 mg/kg Dry Tomato Waste) in comparison with hexane (34.45 mg/kg DTW), ethyl acetate (46.21 mg/kg DTW), acetone (51.90 mg/kg DTW) and ethanol (17.57 mg/kg DTW), therefore it could be a good substitute of “traditional” organic solvents used in lycopene extractions. These extractions were carried out at 70 ℃ except for acetone (50 °C). However, ethyl lactate, even at 25 °C obtained a total yield of 202.73 mg/kg DTW, showing that ethyl lactate extracts more carotenoids at ambient temperature than the other ones at higher temperatures, which would reduce the process energetic cost compared to other solvents. Moreover, in the same study, acetone extracted more effectively tomato carotenoids than ethanol due to better penetration of acetone to plant cells where carotenoids are enclosed [16]. The effect of temperature in carotenoids extraction was also an important parameter. This fact could be related with that higher temperatures promote the destruction of cellular structure and, as a result, to the higher carotenoid content released from the tomato matrix [16]. In addition, the solubility of the material being extracted and its diffusivity increased with temperature, which improves extraction yields.

In another study, the same authors checked the lycopene extraction effectiveness of individual and binary mixtures of hexane, ethanol, ethyl acetate and acetone [68]. In this case, the use of binary mixtures hexane-ethanol and hexane-ethyl acetate, improved the total yield compared with that obtained by any of the individual solvents. In contrast, the acetone alone obtained higher yield than with hexane-acetone. Acetone is a good solvent and a wetting material that penetrates easier in the solid matrix than binary mixture with hexane. Taking into account the carotenoid yield, the best solvent was the binary mixture hexane-ethyl acetate. Thus, the optimization test was carried out with this mixture. In this case, authors concluded that the optimised conditions for maximum carotenoids yield were 45% of hexane in the binary solvent mixture, 1:9 solid/solvent ratio and using 0.56 mm of particle size [68].

As occurred in the previous mentioned studies, the response surface methodology was used by other authors to compare the best conditions for lycopene extractions [69]. These authors used a central composite design with five independent variables [solvent/solid ratio (20:1 to 60:1 *v*/*w*); number of extractions (1–5); temperature (20–60 °C); particle size (0.05–0.43 mm); extraction time (4–20 min)] to study their effects on lycopene extraction. The solvent employed in this study was hexane/acetone/ethanol (2:1:1). The effect of temperature and number of extractions revealed that, with increase in number of extractions and temperature, the lycopene yield increased significantly, while particle size did not affect the lycopene yield [69]. The optimised conditions were 50 °C, 4 extractions of 8 min each, 30:1 solvent/solid ratio and 0.15 mm particle size [69].

In order to maximize the recovery of lycopene, different auxiliary extraction technologies were tested. To this regard, ultrasound-assisted extraction (UAE), high hydrostatic pressure-assisted extraction (HHPE), microwave-assisted extraction (MAE), ultrasound/microwave-assisted extraction (UMAE) and ultrasound under-pressure (UUP) were employed in different researches.

In a very recent research study, authors applied high hydrostatic pressure (HHPE) to tomato pulp and study the influence of solvent mixture and pressure in the extraction yield and lycopene content [70]. In this case, the polar/non-polar solvents varied from 40/60 to 60/40, while the tested pressures ranged between 250 and 450 MPa. Both, yield and lycopene content increased as increased pressure (450 MPa) and non-polar solvent (60% hexane) fraction.

Ultrasound (UAE) can also be used to improve the extraction of lycopene. This technology increase the penetration of solvent into plant cells and favour the disruption of cell walls, which facilitates the release of contents and the contact between solvent and analyte [65,71]. The results obtained in a study carried out comparing conventional organic solvent and UAE, using in both cases hexane/acetone/ethanol, (2:1:1) as solvent, showed that UAE of lycopene required less time, lower temperature and lower solvent than conventional extraction [72]. In conventional extraction, temperature, solid/solvent ratio and time had a significant influence in lycopene extraction, being the optimal lycopene recovery (9.39 mg/100 g) at 60 °C, 1:50 solid/solvent ratio and 40 min of extraction. The highest temperature, extraction time and solvent used in the extraction improved lycopene yields. In the case of UAE, the influence of power, solid/solvent and extraction time in lycopene recovery was tested. The use of UAE allowed these authors obtains similar yields (8.99 mg/100 g) using 90W of UAE power, less solvent (1:35 solid/solvent) and less temperature (5 °C) than conventional extraction [72].

The same findings were also proved in a more recent research [73]. In this case, using the same solvent mixture, the application of UAE increased the lycopene from 5.22 to 7.01 mg/100 g of tomato. Moreover, the authors stated that to achieve an 80% lycopene extraction rate, the ultrasound-assistance (10 min) was much more efficient than the conventional solvent method (20 min) in terms of extraction time. Thus, the results indicated that UAE required shorter time and less solvents consumption than conventional extractions, even at lower temperatures, which allowed extract thermal-sensitive compounds in a more effective way [73]. Moreover, UAE is endowed with the advantages of inexpensiveness, simplicity, reproducibility, and ease of operation during the extraction protocols of myriad bioactive components [78].

On the other hand, the use of UAE was also combined with other auxiliary techniques to improve the release of lycopene. In this sense, the application of biocatalysis (enzyme-assisted extraction with cellulase) improved the lycopene yields both, with and without the application of UAE. Obviously, the biocatalysis-UAE combined extraction resulted in the best yield results, because the benefits of enzymatic-assisted (rupture of membranes and release of cell content) improved the effectiveness of sonication [78]. In other research, authors studied the combination of UAE and pressure (ultrasound under pressure) to improve the carotenoids extraction, using hexane/ethanol as solvent [74]. The results showed an increased in carotenoids yield with the combined techniques, with a maximum yield of 18.3 mg/100 g at 50 kPa pressure, 94 µm of ultrasound amplitude and 6 min of extraction time. After that, using these optimal conditions, authors also tested the influence of solvent (25–75% hexane) and temperature extraction (25–45 °C). In this case, the best conditions were the application of hexane/ethanol (50:50) at 45 °C. Manosonication improved the carotenoids yield from 7.64 mg/100 g (control samples) to 14.08 mg/100 g. According to the results obtained, authors concluded that the temperature and pressure improved the effectiveness of UAE. Thus, manosonication assisted extraction is a promising technology for the carotenoids extraction from tomato by-products at relatively short extraction times [74].

The use of microwave-assisted extraction (MAE) for lycopene extraction from tomato peels was also tested [75]. In this research, a response surface technology was applied to obtain the optimal conditions (solvent, time and microwave power) for the lycopene recovery. The application of MAE resulted in a very low extraction times achieved better results than conventional extractions (45 °C, 30 min). In fact, the optimal conditions for the lycopene yield (13.87 mg/100 g) were the use of ethyl acetate as solvent and the application of 400 W of microwave power during 1 min [75]. This technology was also combined with ultrasound, resulting in ultrasound-microwave assisted extraction (UMAE). In a research study, the application of UAE and UMAE was compared to the lycopene extraction for tomatoes [76]. The influence of power (in UMAE), temperature (in UAE) and time and solid/solvent ratio (in both extraction techniques) were tested. The optimal conditions in UMAE were 98 W of microwave power, 6.1 min of extraction time and 1:10.6 solid/solvent ratio, while in UAE extraction the best lycopene yield was achieved at 86.4 °C, 29.1 min of extraction time and 1:8 solid/solvent ratio. With these results it is easy to conclude that UMAE is the best method, due to the use of UMAE reduced time extraction and improve the lycopene yields in comparison with UAE [76]. This could be explained because selective fast heating of microwave resulted in a physical disruption of tomato cells, which improve the extraction effectiveness [75].

Finally, although the use of different assisted extraction technologies and the optimization of multiple parameters that affect carotenoids extraction presented clear advantages as low extraction times or the use of less solvent amounts, among other, all researchers commented above use toxic solvents to the lycopene extraction. Thus, to overcome this problem, a recent study proposed the utilization of edible oils (renewable and non-toxic solvent) as substitute of toxic organic solvent to recover lycopene from tomato by-product [77]. The green extraction proposed by these authors is the combination of edible oil (sunflower oil) with UAE. The use of oil is a promising substitute to the conventional solvents due to the high solubility of lycopene in oil and acts as barrier against oxygen, delaying the oxidative degradation rate of carotenoid extract [77]. Additionally, the benefits reported above of the use of UAE resulted in improve of lycopene yields. In this study, the response surface methodology was employed to assess the influence of extraction time, ultrasonic intensity and solid/solvent ratio in lycopene yield. The ideal conditions were the application of 70 W/m^2^ during 10 min and the use of 1:5 solid/solvent ratio. According with the results, the use of Oil-UAE at optimal conditions extracted higher amounts of lycopene (91.5 mg/100 g) in 10 min than the conventional extraction, using hexane (63.7 mg/100 g) or hexane/acetone/methanol (2:1:1) mixture (74.9 mg/100 g) after 1 h [77]. However, it should be noted that the use of this technique does not allow the removal of the solvent (oil), so, if the lycopene will be used to reformulate some food must be incorporated together with the oil. This does not have to be an inconvenience, but it must be taken into account when designing the experiment.

### 3.2. Green Technique: Supercritical Fluid Extraction

As commented above, in order to limit the use of large amounts of toxic organic solvents, SFE was proposed as alternative. SFE is environmentally-friendly extraction method that presented a great growth in food industries in the last decade [71]. This technique uses non-toxic organic solvents, which reduces energy use, results in more sustainable processing, and environmental pollution [79]. In SFE, solvents are used close to their critical temperature and pressure to obtain solutes from a liquid or solid matrix under pressurized conditions. In these conditions, the solvents present intermediary characteristics between gases and liquids, which facilitate the recovery of the objective compounds. Carbone dioxide (CO_2_) is the most widely used SFE solvent in food applications since it is generally recognized as safe (GRAS) [71]. CO_2_ has a moderate critical temperature and pressure (31.1 °C and 7.4 MPa) and can be readily removed by a simple pressure reduction [80]. Moreover, the SFE is carried out in the absence of light and oxygen, which reduces the degradation of the compounds. Thus, the use of SFE-CO_2_ is an efficient alternative process to conventional solvent extraction methods, especially for extracting lipophilic plant materials [3]. 

However, the solubility of carotenoids in SFE-CO_2_ is still relatively low compared with to their solubility in organic solvents [68]. The efficiency of SFE process is mostly affected by pressure, extraction temperature, extraction time, CO_2_ density or CO_2_ flow rate [71]. Therefore, optimization of extraction conditions is the most important stage to ensure high extraction yields. Multiple studies were carried out to extract carotenoids and lycopene from tomato and tomato by-product using SFE-CO_2_. In fact, in a recent research the use of SFE-CO_2_ resulted in higher lycopene yield in comparison with conventional solvent extraction [81]. The tomato extracts obtained from SFE-CO_2_ had some advantages, as a higher colour intensity and a more pleasant smell and purity than those from the conventional solvent extractions [62].

The optimal SFE-CO_2_ conditions to recover carotenoids from tomato by-product are shown in Table 2. After reviewed the published articles, the results reported by several papers showed that both, oleoresin and lycopene yields increased with pressure and temperature [81,82,83,84,85,86,87,88,89]. 

It is well known that the CO_2_ density increases with the pressure. In fact, an increase from 33.5 MPa to 45 MPa resulted in an increase in the density about 7.65% [90]. High CO_2_ density increased the solvating power of the supercritical fluid and the molecular interactions between solute and CO_2_, thus, improved the ability of the CO_2_ to solubilize carotenoids [88,91]. At lower densities, the polarity of CO_2_ is more like non-polar solvent (hexane), while with an increment in their density, it is like chloroform. 

In contrast to the pressure effect, a temperature increase reduces the solvent density and consequently reduces the solubility of lycopene, but promotes the transport of solute in the matrix and/or from the matrix into the solvent [87,90,92]. Thus, a balance between these two apparently contradictory effects which results in an overall tendency to improve the carotenoids extraction as the temperature rises [88].

An important point is that the antioxidant activity of lycopene-rich extract showed a significant decrease as increased extraction temperature. However, some authors reported that temperatures between 40–70 °C did not affect antioxidant capacity [87]. In similar way, other study reported that the highest lycopene yield was obtained using 80 °C, but the extract obtained at 40 °C had the strongest antioxidant activity regarding both its free radical scavenging capacity and its singlet oxygen quenching ability [88]. Lycopene in an oleoresin was degraded predominately through oxidation at 25–50 °C, while with temperatures higher than 75 °C the main process is trans-cis isomerization. To this regard, it was reported that approximately 53.5% were degraded after 1 h at 100 °C [87]. In contrast with this results, other study reported that the application of 110 °C during 40 and 50 min did not produce any change in the composition of lycopene isomers [93]. Generally speaking, there is no dramatic isomerization at temperatures lower than 75 °C [82]. With all this in mind, it seems clear that for the correct choice of the extraction temperature there must be a compromise between the oleoresin yield and their antioxidant activity [81].

Both, pressure and temperature have extremely high influence in the effectiveness of carotenoids extraction. However, according different studies, the effect of temperature was stronger than that of pressure in extraction yields [81,82,83].

The CO_2_ flow also influences the extraction process. Some researches pointed out that the higher the supercritical fluid rate was, the higher was the lycopene recovery yield from tomato by-product [81,88], while other did not observe significant differences when the flow increase from 1 to 2 mL/min [87]. However, the use of very high flows produced low yields. This fact is related with the channelling effect, that consists in at high flow rate, the solvent passes around the solid matrix and does not have sufficient residence time to diffuse through the pores within the sample matrix [92]. Other authors related the lower yields at high flows with the reduced amount of time the solvent was in contact with the sample [91].

Finally, the particle size highly affects the extraction recovery. Generally speaking, the smaller the particles of tomato by-product, the better was the extraction recovery [93]. This can be explained by the fact that the particle reduction process causes the rupture of the cell walls and increases the contact surface of the fine particles with the CO_2_ extraction solvent [81,89]. However, the use of very small particle sizes is not recommendable, because it causes packing of extraction bed and results in a channelling effects [89].

In order to enhance the solubility of carotenoids in CO_2_, multiple modifiers and co-solvents were tested. First of all, although both terms are often used interchangeably, modifiers and co-solvents are different. Co-solvents are some substance that is incorporate to the CO_2_ flow and its concentration is constant throughout the process. In contrast, modifiers are similar to co-solvents in that they aid in the extraction, but modifiers are added directly to the sample prior to extraction, instead of with the solvent [91], thus its concentration decreases as the extraction progresses.

The choice of modifiers and/or co-solvents becomes a great challenge. The first aspect is that they must increase the solubility of analyte in the supercritical fluid and must favour the penetration of CO_2_ in the sample matrix in order to enhance the extraction process [82]. Thus, based on the lipophilic properties of lycopene, organic solvents and edible oils were used as co-solvents and modifiers. To this regard, some research concluded that the use of edible oils (hazelnut oil [90] or olive oil [82,95]) as modifiers improved the carotenoids recovery from tomato and tomato by-products. However, not only the edible oils enhance lycopene extraction. In a study, the combination of ethanol and olive oil obtained the best lycopene yields [82], while other authors concluded that the use of water miscible solvents (acetone of methanol) gave higher recoveries of lycopene than ethanol, while the use of immiscible water solvents (hexane of dichloromethane) gave the lowest yields [93]. The main drawback of the use of organic solvents is that they could remain in trace amounts in the final extract, making it unsuitable for its use in food industry [85,94]. 

In another study, authors proposed the co-extraction of lycopene from tomato by-product and oil from hazelnuts powder [94]. This fact allows them to increase the hazelnut oil (as powder) in sample matrix without losing rheological properties in comparison with a previous study using direct addition of hazelnut oil as modifier [90]. These authors found that using co-extraction lycopene-oil adding hazelnut as powder increased lycopene yield and reduced the extraction time in comparison with the direct addition of hazelnut oil, which has considerable energetic and economic benefits [94].

Other authors compared the influence of both, ethanol and canola oil as co-solvents [92]. In this case, the results showed a significant increase in lycopene extraction using co-solvents in comparison with pure CO_2_. Additionally, it is also important to note that canola oil is a better co-solvent than ethanol.

Thus, the optimization of SFE-CO_2_ extraction conditions is so important to maximize the carotenoid recoveries that maintain a high antioxidant capacity. Moreover, in order to increase the solubility of carotenoids in CO_2_, the promising use of edible oils as co-solvents or modifiers allows greater recoveries even than with toxic solvents.

## 4. Use of Tomato in Meat Products

The advantages of the use of tomato and tomato by-products in the meat industry are mainly their excellent antioxidant properties due to their composition, rich in bioactive compounds and their intense red colour, which allow to replace synthetic antioxidants and colourants by tomato powder, paste, oleoresin or extract. Moreover, lycopene presents multiple advantages that make it ideal for application in the food industry, such as its stable to heat and extreme pH values, effective in low concentrations, has no off-flavours and covers the full range of colours (yellow-orange-red) [60]. In this sense, multiple researches reformulated different meat products and included tomato or tomato extracts in their composition (Table 3).

The use of both, oleoresin and pulp tomato powder were employed for stabilization of colour and oxidative degradation of beef patties [96]. The lipid oxidation was significantly reduced by the addition of oleoresin at 0.55 g/kg and 2 g/kg until 12 days of storage under refrigeration, being the most intense protective effect at highest dose, which indicated that the effectiveness of oleoresin in lipid oxidation prevention is dose-dependent. In contrast, the use of tomato powder exerted very low antioxidant effect in comparison with control, at either of the concentrations used (15 and 50 g/kg). These results demonstrated the antioxidant activity of high amounts of lycopene (higher in oleoresin than in tomato powder), which confirmed the protective effect against oxidative damage. The use of 2 g/kg of oleoresin gave rise to the highest redness, followed by oleoresin (0.55 g/kg) and tomato powder (50 g/kg). However, redness in samples with tomato powder at 15 g/kg did not differ from the control samples. Additionally, the use of oleoresin or tomato powder (50 g/kg) delayed the discolouration process in comparison with control samples during refrigeration storage. In accordance with instrumental colour, the sensory analysis also showed that the application of oleoresin and tomato powder delayed both, discolouration and off-flavour formation during storage at refrigeration. 

The use of tomato powder in pork patties [11] and beef burgers [63] and tomato paste in beef patties [19] were also tested by other authors. Regarding lipid oxidation, the reformulation of patties with tomato paste at all levels tested (5, 10 and 15%) and tomato powder, also in all levels (0.25, 0.5, 0.75 and 1%) resulted in a significant lower thiobarbituric acid reactive substances (TBARS) values than control samples during refrigerated storage. Additionally, both studies observed that the antioxidant activity was dose-dependent, which could be attributed to lycopene’s activity. In a similar way, the redness of samples containing tomato powder [11,63] and tomato paste [19] was higher while the discolouration rate during storage was lower than control samples. The colour improving effect can be attributed to the lycopene (red colour) present in tomato paste and powder and its antioxidant effect. This increase in redness in reformulated patties could be more attractive to consumers than the control. In fact, the results obtained in sensory analysis showed highest colour scores in patties with tomato powder [11] and did not show differences between control and the samples treated with tomato paste [19]. In contrast, the addition of tomato powder in beef burgers resulted in a low colour scores than control, probably due to an increase in the red/orange tone of this samples produces a colour very different from that expected in a conventional hamburger [63]. The flavour scores and overall acceptability increased as tomato powder was included in pork patties [11].

Contrary with the aforementioned research, in a recent study, the addition of tomato extract to lamb patties did not produce any effects [5]. In this case, the discolouration rate and the lipid oxidation in samples with tomato extract did not differ from control samples, thus no antioxidant effect of tomato extract was observed in this study. In material and methods section, these authors explained that the tomato extract was prepared with acidified water. As commented above, carotenoids and especially lycopene are immiscible in water, which can help us to understand the lack of antioxidant activity of the extract used in this study.

Other meat products that were reformulated with tomato and studied by several researchers were frankfurters and cooked sausages. In all cases, the addition of tomato powder [11,20,97,98] or tomato paste [2,99] enhanced the redness of frankfurter and cooked sausages and delayed the discolouration during storage [98]. In contrast, the antioxidant effect of the tomato powder or paste showed different trends among the studies. The addition of tomato paste at 12% in the frankfurters resulted in an increase of lipid oxidation (TBARS) [2]. In similar way, the addition of tomato powder at 4%, also in frankfurters, resulted in a significant increase of TBARS values, while the addition of 2% did not affect lipid oxidation in comparison with control sausages [20]. In cooked sausages the addition of tomato powder did not show influence in the first 21 storage days, while at day 28, the addition of powder resulted in a potent inhibition of lipid oxidation, which demonstrate that the tomato powder retarded lipid oxidation until the end of storage [100]. Similarly, the addition of tomato powder [98] and tomato paste [99] to cooked sausages reduced significantly the lipid oxidation. Additionally, it was observed that oxidation decreased as the tomato level increased, which demonstrate that the antioxidant effectiveness of tomato is dose-dependent [98].

In regard to sensory analysis, the addition of tomato powder and paste did not affect or improve the sensory properties of frankfurter and cooked sausages [97,98,99]. In general, colour scores of samples containing tomato were more acceptable by consumers [20,97]. Moreover, the tomato inclusion increased the acceptability of sausages, even when its addition promoted lipid oxidation [20,97,98].

In addition to cooked sausages, it also tested the influence in dry-fermented sausages [18], showing practically the same trend. Redness was significantly higher in sausages reformulated with tomato at the end of the ripening process (after 21 days). The sensory characteristics did not show significant differences between control and treatment samples and all samples presented good overall acceptability [18].

On the other hand, the use of tomato powder, tomato paste and crystalline lycopene in beef minced meat [101] and “in natura”, different wastes and final product of industrial tomato process in high-pressure processing minced chicken meat [102] were tested. In the study carried out with beef minced meat, the use of crystalline lycopene (without the addition of nitrite) showed the highest redness at the initial point (day 0) and the lowest lipid oxidation until day 8 of storage (measured as peroxide value), which indicate that lycopene act as good colourant and antioxidant in meat [101]. However, in the same study, the samples with tomato paste presented the most stable and the smallest increase in peroxide value during all refrigerate storage, which also demonstrate the antioxidant effectiveness of tomato by-products [101]. The use of tomato and tomato wastes in minced chicken meat showed different effects, depending on the high-pressure and the tomato product applied to the meat [102]. In the samples treated with 600 MPa, a lag phase of 6 days was identified in lipid oxidation (TBARS) with the use of both, tomato waste and final product. In a similar way, although the use of 800 MPa efficiently induced lipid oxidation, lower rate of development of secondary oxidation products was observed for the meat with the tomato waste addition, which indicates that tomato waste was a good antioxidant [102]. 

The higher antioxidant activity of tomato waste (composed by skin and seeds) than of the other products could be related with the fact that this product had the highest phenolic and flavonoids amounts [102]. In fact, author pointed out those specific flavonoids found in higher amounts in wastes, as rutin, quercitrin and naringenin may be more efficient as antioxidant than carotenoids with respect to preventing lipid oxidation in pressurized chicken meat. Regarding colour, very small amounts of tomato products were added to the meat samples (between 0.1 and 0.3%) in order to not contribute to the colour of the meat, thus no differences were observed among samples [102].

In addition to the cooked sausages discussed above, the effect of reformulation with tomato of other pork cooked meat products, as mortadella [9] and luncheon roll [10] were also analysed. In the case of mortadella, three groups of products were developed with 2, 6 and 10% of tomato paste [9]. Regarding lipid oxidation, although in the initial steps of meat-product manufacture the reformulation did not show differences among batches, the TBARS values of samples containing 10% of tomato paste showed the lowest values (31.8 mm MDA/100 g), followed by 6% samples (36.4 mm MDA/100 g) and finally the highest TBARS values (37.8 mm MDA/100 g) were observed in samples containing the lowest values of tomato (2%) [9]. This fact seems to indicate that, despite not being significant, the application of tomato shows a tendency to reduce oxidation in this product. This is confirmed by observing the evolution of lipid oxidation during mortadella shelf-life. Samples with tomato paste addition had good oxidative stability compared with control. It is also important to note that in this study no differences were observed between the three levels of addition, so it seems that the antioxidant activity in this case was not dose-dependent [9]. Additionally these benefits must be included that the reformulation of mortadella with tomato paste significantly improved the colour intensity and texture scores and these products received more extremely positive responses than control samples [9].

In luncheon roll, the addition of tomato powder (1.5 and 3%) acted as colourant agent, which resulted in an increase in the redness of the raw meat (day 0) and cooked pork luncheon rolls on each storage day [10]. In contrast to the results obtained in the mortadella study, the use of tomato peel powder increased lipid oxidation in luncheon rolls. However, none of the luncheon samples exceeded 0.41 mg MDA/kg, indicating a low level of rancidity over the 14 days of storage [10]. The tomato powder addition also resulted in a decrease in overall acceptability and colour attributes, which is related with the orange/red colour tone, very different from that expected in a conventional product, as the amount of tomato increase. Thus, the detrimental physicochemical and sensory properties indicated that the production of rolls enriched with tomato powder would not be feasible [10].

As we have discussed in this section, there are multiple studies in which meat products were reformulated with tomato as natural antioxidant/colourant. Among the studies there was great variability in the results obtained, probably due to the fact that some authors used tomato directly (as powder or paste), while others used extracts or extracted oleoresins. It is also well known that the extraction conditions as well as the previous treatments such as drying conditions for the tomato by-product, light exposure, grinding and tomato cultivar significantly affect the lycopene content as well as its antioxidant capacity and isomerization processes. Due to these factors, carotenoid extraction/utilization immediately after processing or with minimal storage time after drying would be recommend to minimize losses [15]. With this in mind, the great diversity of researches and conditions in which these are carried out will also determine the variation in the results obtained between them. In spite of all this, the results obtained in many studies indicate that the use of tomato in meat offers promising results.

In a general conclusion, lycopene-enriched tomato products, such powders, paste, oleoresin or extracts may be envisaged as natural antioxidants and colourants for use in meat and meat products [96].

## 5. Conclusions

Tomato processing produces huge amounts of by-products, which contains several bioactive compounds. Thus, tomato by-products are a renewable and potential source of natural additives, which could be used in food industries, following the demands of consumers (natural additives) and improving waste management. In fact, lycopene, the most important carotenoid in tomato is widely used by the food industry as antioxidant and colorant.

Due to the lipophilic character of carotenoids, during decades the use of toxic organic solvent was the unique way to obtain carotenoid rich oleoresin from tomato by-products. To solve the environmental and health implications and problems of the use of toxic solvents, supercritical fluid extraction or the use of edible oils as a solvent in conventional extractions are two promising and proven methods to obtain carotenoids rich extracts. The extraction conditions must be carefully selected, to obtain high yields and at the same time maintain a high antioxidant capacity. Therefore, the use of auxiliary techniques (ultrasound, high hydrostatic pressure, microwave and their combinations) in conventional extractions and the use of co-solvents and modifiers in supercritical fluid extraction improve the yields and, in general, reduce extraction times and temperature, which reduce costs and protect thermo-labile compounds.

The number of studies conducted with application of tomato by-products in the meat industry is reduced, compared to the huge amount of studies carried out with other plant extracts obtained with aqueous or hydro-alcoholic solutions. This fact could be related to the complexity and the use of toxic organic solvents to obtain oleoresins rich in lycopene, compared to the ease of obtaining aqueous extracts rich in polyphenols. 

Thus, the use of green extraction techniques to obtain lycopene-rich oleoresin, the control of all parameters that could affect the oleoresin antioxidant capacity and the lycopene isomerization process and their application in meat industry must be more studied. Promising results obtained in different studies suggest that the use of tomato by-products as natural additives can be used to extend the shelf-life of meat products providing the consumer with food that contains only natural additives. Their use improved nutritional quality, reduced lipid oxidation and increased stability during the shelf-life period of meat products, while retaining or increasing sensory properties and overall acceptability. Additionally, new functional meat products would be developed, due to the human health benefits of lycopene intake. Thus, considering the positive effects of tomato on human health, the effect of meat products reformulated with tomato on health should also be investigated.

## Figures and Tables

**Table 1:** Extraction conditions and organic solvents applied in recovering carotenoids from tomato by-products.

Material	Solvent	T (°C)	Time (min)	S/S Ratio ^1^	Auxiliary Technique	Yield	Ref.
Skin	Hexane/acetone/ethanol (2:1:1)	50	8 (×4)	1:30	-	1.99 ^a^	[69]
Skin + seeds	Hexane	70	30	1:10	-	3.45 ^b^	[16]
	Acetone	50				5.19 ^b^	
	Ethanol	70				1.76 ^b^	
	Ethyl acetate					4.62 ^b^	
	Ethyl lactate					24.3 ^b^	
Skin + seeds	Ethanol	25	30	1:10	-	0.61 ^b^	[68]
	Hexane					2.52 ^b^	
	Ethyl acetate					3.15 ^b^	
	Acetone					3.34 ^b^	
	Hexane/ethanol (50:50)					2.81 ^b^	
	Hexane/acetone (50:50)					3.05 ^b^	
	Hexane/ethyl acetate (50:50)					3.65 ^b^	
	Hexane/ethyl acetate (45:55)			1:9		3.75 ^b^	
Pulp	Hexane/ethanol/acetone (60:20:20)	-	24 h	1:2	-	0.36 ^a^	[70]
		20	10		HHPE (450 MPa)	2.01 ^a^	
Skin + seeds	Hexane/acetone/ethanol (2:1:1)	60	40	1:50	-	9.39 ^a^	[72]
		5	30	1:35	UAE (90W)	8.99 ^a^	
Skin + seeds	Hexane/acetone/ethanol (2:1:1)	15	30	1:35	-	5.72 ^a^	[73]
					UAE (90W)	7.69 ^a^	
Skin + seeds + pulp	Hexane/ethanol (50:50)	45	6	1:33	UUP Manosonication (50kPa/US amplitude 94 µm)	14.08 ^b^	[74]
Peel	Ethyl acetate	-	1	1:20	MAE (400W)	13.87 ^a^	[75]
Skin + seeds + pulp	Ethyl acetate	86.4	29.1	1:8	UAE (50W)	89.4% ^c^	[76]
		-	6.1	1:10.6	UMAE (98W)	97.4% ^c^	
Skin + pulp	Sunflower oil	-	10	1:5	UAE	91.5 ^a^	[77]
	Hexane		60		-	63.7 ^a^	
	Hexane/acetone/methanol (2:1:1)				-	74.9 ^a^	

T: Temperature; ^1^ Solid/Solvent ratio; - data not available or auxiliary technique not used; HHPE: high hydrostatic pressure extraction; UAE: Ultrasound-assisted extraction; UMAE: ultrasound/microwave-assisted extraction; UPP: ultrasound under-pressure; MAE: microwave-assisted extraction; ^a^ mg lycopene/100 g; ^b^ mg carotenoids/100 g; ^c^ % of total lycopene.

**Table 2:** SFE-CO_2_ conditions applied in recovering carotenoids from tomato by-products.

Material	Pressure (MPa)	T (°C)	Time (min)	Flow	Particle Size (mm)	Modifier/Co-Solvent	Yield	Ref.
Tomato juice	35	80	180	1.7 g/min	-	-	76.9% ^a^	[88]
Skin + seeds	30	60	-	0.59 g/min	0.36	-	-	[89]
Skin + seeds	34.5	86	20	2.5 mL/min	-	-	61% ^a^	[91]
Skin + seeds	40	70	90	2 mL/min	1	-	19.21 ^b^	[87]
Skin	41	80	105	4 g/min	0.3	-	72.8 ^c^	[81]
Skin + seeds	30	80	-	13.2 g/min	0.345	-	80% ^a^	[84]
Skin + seeds	46	80	22.7	2 mL/min	-	-	90.1% ^a^	[83]
Tomato juice + pulp	53.7	73.9	155	-	<0.2	-	25.12 ^d^	[86]
Skin + pulp	27.6	80	30	500 cm^3^/min	-	-	64.41 ^c^	[85]
Whole tomato	40	40	360	0.5 L/min	0.5–1	-	0.14 ^e^	[92]
						Ethanol *	0.23 ^e^	
						Canola oil *	0.57 ^e^	
Whole tomato	40–45	60–70	240	10 kg/h	-	Hazelnut powder ^+^	72% ^a^	[94]
Skin	35	75	-	3.5 L/min	-	Ethanol (10%) olive oil (10%) ^+^	73.3 ^b^	[82]

T: Temperature; - data not available or modifier/co-solvent not used; ^+^ Modifier; * Co-solvent; ^a^ % of total lycopene; ^b^ µg lycopene/g; ^c^ mg lycopene/100 g; ^d^ g oleoresin/100 g; ^e^ mg lycopene.

**Table 3:** Meat products reformulated with tomato by-products.

Meat Product	Material	Amount	Main Effects	Ref.
Patties & burgers	Tomato Oleoresin	0.55 g/kg	↓ Lipid oxidation & discolouration; ↑ Redness	[96]
		2 g/kg		
	Tomato Powder	15 g/kg	No effects	
		50 g/kg	↓ Lipid oxidation & discolouration; ↑ Redness	
	Tomato Paste	5, 10 & 15%	↓ Lipid oxidation & discolouration; ↑ Redness; = Sensory colour scores	[19]
	Tomato Powder	1.5, 3, 4.5 & 6%	↓ Discolouration; ↑ Redness; ↓ Sensory scores	[63]
		0.25, 0.5, 0.75 & 1%	↓ Lipid oxidation & discolouration; ↑ Redness; ↑ Sensory properties	[11]
	Aqueous Extract	1 g/kg	No effects	[5]
Frankfurter and cooked sausages	Tomato Powder	1 & 2%	↓ Lipid oxidation; ↑ Redness	[100]
		2 & 4%	↑ Lipid oxidation; ↑ Redness; ↑ Sensory properties	[20]
		0.8, 1.2 & 1.5%	↓ Lipid oxidation; ↑ Redness; ↑ Sensory properties	[98]
		1, 3, 5 & 7%	↑ Redness; ↑ Sensory properties	[97]
	Tomato Paste	2.5 & 3%	↓ Lipid oxidation; ↑ Redness; ↑ Sensory colour scores	[99]
		2, 4, 6, 8, 10, 12 & 16%	↑ Lipid oxidation; ↑ Redness; ↑ Sensory colour scores	[2]
Dry-fermented sausage	Tomato Powder	6, 9 & 12 g/kg	↑ Redness; = Sensory properties	[18]
Minced meat	Tomato and tomato waste	0.1-0.3%	↓ Lipid oxidation; = Redness	[102]
	Tomato Powder, Paste and crystalline lycopene	-	↓ Lipid oxidation & discolouration; ↑ Redness	[101]
Luncheon roll	Tomato Powder	1.5 & 3%	↑ Lipid oxidation; ↑ Redness; ↓ Sensory properties	[10]
Mortadella	Tomato Paste	2, 6 & 10%	↓ Lipid oxidation; ↑ Redness; ↑ Sensory properties	[9]

↓ Decrease; ↑ Increase; = no significant changes.
